# Slow Transition to Low-Dimensional Chaos in Heavy-Tailed Recurrent Neural Networks

**Published:** 2025-10-24

**Authors:** Eva Yi Xie, Stefan Mihalas, Łukasz Kuśmierz

**Affiliations:** 1 Allen Institute, Seattle, WA, USA; 2 Princeton Neuroscience Institute, Princeton University, NJ, USA

## Abstract

Growing evidence suggests that synaptic weights in the brain follow heavy-tailed distributions, yet most theoretical analyses of recurrent neural networks (RNNs) assume Gaussian connectivity. We systematically study the activity of RNNs with random weights drawn from biologically plausible Lévy alpha-stable distributions. While mean-field theory for the infinite system predicts that the quiescent state is always unstable—implying ubiquitous chaos—our finite-size analysis reveals a sharp transition between quiescent and chaotic dynamics. We theoretically predict the gain at which the finite system transitions from quiescent to chaotic dynamics, and validate it through simulations. Compared to Gaussian networks, finite heavy-tailed RNNs exhibit a broader gain regime near the edge of chaos, namely, a slow transition to chaos. However, this robustness comes with a tradeoff: heavier tails reduce the Lyapunov dimension of the attractor, indicating lower effective dimensionality. Our results reveal a biologically aligned tradeoff between the robustness of dynamics near the edge of chaos and the richness of high-dimensional neural activity. By analytically characterizing the transition point in finite-size networks—where mean-field theory breaks down—we provide a tractable framework for understanding dynamics in realistically sized, heavy-tailed neural circuits.*

## 1 Introduction

Advances in connectomics yield increasingly detailed wiring diagrams of neural circuits across species and brain regions [Dorkenwald et al., 2024, The MICrONS Consortium, 2025]. This progress raises fundamental questions: what structural principles govern neural circuits, and how do they support the brain’s remarkable computational power? One such prominent structural feature is the presence of heavy-tailed [Foss et al., 2011] synaptic weight distributions, consistently observed across the mammalian cortex [Song et al., 2005, Lefort et al., 2009, Dorkenwald et al., 2022], mammalian hippocampus [Ikegaya et al., 2013], and even in the *Drosophila* central brain [Scheffer et al., 2020]. Notably, this feature stands in sharp contrast to the Gaussian weight assumptions that dominate theoretical neuroscience studies and light-tailed distributions utilized in the standard initialization schemes in modern artificial neural networks [LeCun et al., 2002, Glorot and Bengio, 2010, He et al., 2015]. One way to formally model heavy tails is with the family of Lévy α-stable distributions [Feller, 1971, Borak et al., 2005], which emerges as a natural generalization of the familiar Gaussian distribution (α=2) via the generalized central limit theorem. The family is parameterized by a stability index α, where smaller values of α correspond to heavier tails. For α<2, these distributions feature heavy, power-law tails. Similarly to experimentally measured synaptic weights, samples generated from such distributions consistently contain large outliers that dominate many sample statistics. As we show in this paper, this can strongly affect neural dynamics.

One key phenomenon studied in theoretical neuroscience and machine learning is the transition to chaos, which has long been hypothesized to support optimal information flow and computational capacity at the so-called *edge of chaos* in a wide variety of randomly initialized neural networks. This encompasses RNNs [Bertschinger et al., 2004, Legenstein and Maass, 2007, Toyoizumi and Abbott, 2011, Schuecker et al., 2018] and feedforward neural networks [Schoenholz et al., 2016, Poole et al., 2016]. In particular, Schoenholz et al. [2016] shows that the trainability of deep networks depends on initializing near the edge of chaos: the farther from this critical regime, the shallower a network must be to remain trainable. Similarly, Bertschinger et al. [2004] shows that only near the edge of chaos can RNNs perform complex computations on time series.

In the context of feedforward networks, this effect can be understood in terms of the neural network Gaussian process kernel [Neal, 1996]: outside of the critical point, analogous to the edge of chaos in RNNs, a trivial fixed point with kernel constant almost everywhere is approached exponentially fast, limiting the effective depth of information propagation and network trainability [Schoenholz et al., 2016, Lee et al., 2017]. The transition between chaotic and non-chaotic dynamics in RNNs is often discussed in terms of eigenvalues of the weight matrix [Rajan and Abbott, 2006, Aljadeff et al., 2015]. According to the circular law [Girko, 1985, Tao et al., 2010], its eigenvalues are bounded in a circle of radius proportional to the standard deviation of the distribution of weight entries. If the standard deviation is large enough, some eigenvalues fall outside of the unit circle and the quiescent state becomes unstable, paving the way for chaos to emerge. In contrast to random matrices with light-tailed elements, random matrices with α-stable entries feature an unbounded limiting density of eigenvalues [Bordenave et al., 2011]. In infinite networks, this can lead to the lack of transition between quiescent and chaotic states, with any perturbation ultimately expanding in a chaotic manner [Kuśmierz et al., 2020].

Understanding the computational implications of heavy-tailed recurrent connectivity is especially timely as RNNs have become central to neuroscience modeling. They are used to reproduce latent trajectories from neural recordings [Sussillo and Abbott, 2009, Rajan et al., 2016, Pandarinath et al., 2018, Keshtkaran et al., 2022], to simulate circuit mechanisms of cognitive tasks [Yang et al., 2019, Driscoll et al., 2024], and to complement experiments through hypothesis generation [Pinto et al., 2019, Pagan et al., 2025]. In NeuroAI, RNNs have been embedded into deep reinforcement learning agents to recapitulate biological navigation codes of grid cells [Banino et al., 2018]. Yet, despite their prevalence, theoretical understanding of RNNs with biologically realistic, heavy-tailed weights remains limited.

To that end, the contribution of this paper is four-fold:

- We reveal that finite-size heavy-tailed RNNs exhibit a sharp transition from quiescence to chaos, *in contrast* to the mean-field prediction of ubiquitous chaos in infinite networks with tanh-like activation functions [Kuśmierz et al., 2020].
- We derive theoretical predictions for the critical gain at which this transition occurs as a function of network size, and validate them through simulations.
- We show numerically that heavier-tailed RNNs exhibit a slower transition to chaos, sustaining edge-of-chaos dynamics over a broader gain regime and offering greater robustness to gain variation; we show this can translate to improved information processing, as evidenced by the superior performance of heavy-tailed RNNs on a simple reservoir-computing task.
- We quantify attractor dimensionality as a function of tail heaviness, uncovering a tradeoff between robustness and dynamical complexity: heavier tails compress activity onto lower-dimensional manifolds.

## 2 Related Works

In his seminal work, Neal [1996] examined Bayesian inference in neural networks and demonstrated that, in the infinite-width limit, shallow feedforward networks with standard Gaussian weight initializations converge to Gaussian processes. He noted that this convergence breaks down when weights are drawn from Lévy α-stable distributions, hypothesizing that such heavy-tailed initializations give rise to a richer class of priors beyond the representational capacity of Gaussian process kernels. This insight has since been extended and formalized by a recent series of theoretical works that rigorously characterize the infinite-width limit of feedforward networks, showing convergence to α-stable processes [Favaro et al., 2020, Jung et al., 2021, Bordino et al., 2023, Favaro et al., 2023]. Additionally, in [Favaro et al., 2024], the training dynamics of shallow feedforward networks with heavy-tailed distributions of weights are characterized through the neural tangent kernel [Jacot et al., 2018]. While these studies focus on feedforward architectures, our work complements them by uncovering and characterizing a distinct transition in heavy-tailed feedforward networks via an annealed analysis, an effect not previously reported. We then extend this investigation to recurrent networks.

The critical behavior of heavy-tailed networks has also been examined in both RNNs and feedforward settings. Wardak and Gong [2022] report an extended critical regime in heavy-tailed RNNs, while Qu et al. [2022] demonstrate that a similar extended critical regime emerges in heavy-tailed feedforward neural networks, with training via stochastic gradient descent being most efficient in the region of the parameter space corresponding to the critical regime. Our findings are consistent with these observations and advance them by: (a) explaining the extended critical regime in terms of the behavior of the maximal Lyapunov exponent, (b) showing that the location of the transition depends on the network size, and (c) identifying a tradeoff between the size of the critical regime and the dimensionality of the neural manifold in the critical regime.

Additionally, a mean-field theory of Cauchy RNNs (*i.e.,* with weights following a Lévy α-stable distribution where α=1), is presented in Kuśmierz et al. [2020]. Specifically, they show that Cauchý RNNs with a binary activation function exhibit transition to chaos and generate scale-free avalanches, similarly observed in biological neural recordings [Beggs and Plenz, 2003] and often presented as evidence supporting the critical brain hypothesis [Muñoz, 2018]. Notably, Kuśmierz et al. [2020] note that Cauchy networks with a wide class of activation functions, including tanh studied in our work, are always chaotic in the infinite-size limit, and, as such, do *not* exhibit a transition to chaos. In contrast, our results reveal that this observation is no longer true in the *finite* networks, highlighting the importance of finite-size effects.

Finally, our work complements recent studies on brain-like learning with exponentiated gradients [Cornford et al., 2024], which showed that such updates naturally give rise to log-normal connectivity distributions. Within this broader context, our results offer a theoretical perspective that elucidates the dynamical consequences of these heavy-tailed structures.

## 3 Methods

### 3.1 Setup of recurrent neural network

We study recurrent neural networks that evolve in discrete time according to the update rule

(1) $$x_{i}(t+1)=\phi \left(\sum_{j=1}^{N}W_{ij}x_{j}(t)+I_{i}(t)\right),$$

where ϕ=tanh is the activation function, $I_{i}(t)$ is the external input to neuron i at time t, and N is the number of neurons. The synaptic weights $W_{ij}$ are independently drawn from a symmetric Lévy α-stable distribution [Feller, 1971, Borak et al., 2005], i.e. $W_{ij}\sim L_{\alpha }(\sigma )$ with characteristic function

(2) $$\phi_{L_{\alpha }(\sigma )}(k)=\exp\left(-|\sigma k{|}^{\alpha }\right),$$

and with scale parameter $\sigma =g/N^{1/\alpha }$, where the gain g acts as the control parameter in our analysis. The stability parameter α∈(0,2] affects the tails of the distribution. For α<2, the corresponding density function features heavy, power-law tails, i.e. $\rho_{L_{\alpha }(\sigma )}(x)\propto |x{|}^{-1-\alpha }$ when |x|≫1. The remaining case of α=2 corresponds to the familiar Gaussian distribution with light tails.

We perform analyses on both autonomous (zero-input) and stimulus-driven RNNs. In the latter case (see Appendix E), inputs at each time step are sampled i.i.d. from a Gaussian distribution with zero mean and variance 0.01. This enables us to study how stochastic drive interacts with heavy-tailed synaptic weight distributions to modulate network stability.

### 3.2 Setup of feedforward networks

Although the weight matrix remains fixed during RNN evolution, in our mathematical analysis, we assume that $W_{ij}$ is redrawn at each time step. With such an *annealed* approximation [Derrida and Pomeau, 1986], evolving the RNN for T steps effectively corresponds to passing an input (initial condition) through a feedforward network of T layers. In this case, we can reformulate the update equation (1) as

(3) $$x_{i}^{\left(t+1\right)}=\phi \left(\sum_{j=1}^{N_{t}}W_{ij}^{\left(t\right)}x_{j}^{\left(t\right)}+I_{i}^{\left(t\right)}\right),$$

where $W_{ij}^{\left(t\right)}$ is the $N_{t+1}\times N_{t}$ weight matrix at layer t. In this case, the initial condition $x_{i}^{\left(0\right)}$ is interpreted as the input, and activity at t=T as the output of a T-layer network. Additional inputs could also be passed directly to each layer via $I_{i}^{\left(t\right)}$. Note that we assumed that each layer may have a different width $N_{t}$. The case when $\forall_{t}N_{t}=N$ corresponds to the annealed approximation of (1). We use ${\langle \cdot \rangle }_{X}$ to denote the expected value with respect to a random variable X.

### 3.3 Computation of Lyapunov exponents

To quantify the dynamical stability of RNNs, we compute their Lyapunov exponents across a range of weight scales (gains) g. This also provides an estimate of the maximum Lyapunov exponent (MLE, $\lambda_{\max}$), which measures the average exponential rate at which nearby trajectories diverge in phase space. A positive $\lambda_{\max}$ indicates chaotic dynamics, while a negative value implies convergence to a stable fixed point or limit cycle. When $\lambda_{\max}\approx 0$, the system operates at the *edge of chaos*, a critical regime where perturbations neither grow nor decay rapidly.

We adopt the standard QR-based algorithm [Von Bremen et al., 1997] described in Vogt et al. [2022] (detailed in Appendix C) to compute the Lyapunov spectrum. For each input sequence, we track how infinitesimal perturbations evolve under the hidden-state Jacobians. These perturbations are orthonormalized via QR decomposition at each step, and the logarithms of the diagonal entries of the R matrix are accumulated to estimate the exponents. To avoid transient effects, we include a short warmup period during which the network state evolves but Lyapunov exponents are not accumulated. The MLE is then averaged over multiple random input sequences to obtain a robust estimate.

### 3.4 Participation ratio and Lyapunov dimension

#### Two notions of dimensionality

We analyze the dimensionality of RNN dynamics from two complementary perspectives. The first, based on Lyapunov exponents, quantifies how many directions exhibit local expansion under small perturbations; this is captured by the Lyapunov (Kaplan–Yorke) dimension $D_{\text{KY}}$, derived from the leading part of the Lyapunov spectrum. The second, based on the participation ratio (PR), measures how many orthogonal directions the network activity spans at steady state, using second-order statistics of the hidden states. Intuitively, PR is a linear method that approximates the manifold by an ellipsoid, and as such it may significantly overestimate the dimensionality of a highly nonlinear manifold. In contrast, $D_{\text{KY}}$ is a nonlinear measure that, for typical systems, correctly estimates the information (fractal) dimension of a chaotic attractor [Ott, 2002].

#### Lyapunov dimension

To measure intrinsic dynamical complexity, we compute the full Lyapunov spectrum using the standard QR method (see Section 3.3). Let $\lambda_{1}\geq \lambda_{2}\geq \cdots \geq \lambda_{N}$ be the ordered Lyapunov exponents. Define k as the largest index such that $\sum_{i=1}^{k}\lambda_{i}\geq 0$. Then the Lyapunov dimension [Frederickson et al., 1983, Farmer et al., 1983, Ott, 2002] is defined as:

(4) $$D_{\text{KY}}:=k+\frac{\sum_{i=1}^{k}\lambda_{i}}{\left|\lambda_{k+1}\right|}.$$

Near the edge of chaos, all positive Lyapunov exponents are close to 0 and perturbations along the corresponding directions expand with slow timescales. As a result, in this regime, a higher $D_{\text{KY}}$ indicates that the system evolves on a higher-dimensional *slow manifold* [Krishnamurthy et al., 2022], with more modes contributing to long-term variability and slow divergence–implying a greater capacity to support rich, temporally extended computations. We track all orthogonal directions and update them with QR decomposition at each step after a fixed warmup. We examine how $D_{\text{KY}}$ evolves with gain g across all dynamical regimes.

#### Participation ratio

Let $x^{\left(t\right)}\in {\mathbb{R}}^{N}$ be the hidden state of the RNN at time t, recorded over the final K steps of a length-T trajectory at fixed gain g with K>N, after discarding the initial T−K warmup steps. We compute the empirical covariance matrix $S=\frac{1}{T-1}\sum_{t=1}^{T}\left(x^{\left(t\right)}-\bar{x}\right){\left(x^{\left(t\right)}-\bar{x}\right)}^{⊤}$, where $\bar{x}=\frac{1}{T}\sum_{t=1}^{T}x^{\left(t\right)}$. Let $\tilde{\lambda }$ denote the eigenvalues of S. The participation ratio is defined as [Kramer and MacKinnon, 1993, Gao et al., 2017, Recanatesi et al., 2022]:

(5) $$\mathrm{PR}:=\frac{{\left(\sum_{i}{\tilde{\lambda }}_{i}\right)}^{2}}{\sum_{i}{\tilde{\lambda }}_{i}^{2}}.$$

PR ranges from 1 (all variance in one mode) to N (uniform variance), and quantifies how many orthogonal directions carry substantial variance regardless of stability. It has been widely used to characterize neural dimensionality in biological and artificial circuits [Gao et al., 2017, Recanatesi et al., 2022]. We compute PR across all types of regimes for the post-warmup steady-state trajectories.

## 4 Results

### 4.1 Finite heavy-tailed networks exhibit a predictable quiescent-to-chaotic transition

#### 4.1.1 Information propagation in feedforward networks

We study networks without external inputs $\left(I^{\left(t\right)}=0\right)$. Since ϕ(0)=0, the quiescent state is a fixed point of both (1) and (3). In our mathematical derivation, we focus on the simpler case of annealed dynamics. To study the stability of the quiescent state, we expand (3) around $x^{\left(t\right)}=0$ and obtain a linear equation

(6) $$\varepsilon^{\left(t+1\right)}=W^{\left(t\right)}\varepsilon^{\left(t\right)}$$

where we used $\phi^{\prime }(0)=1$. Since sequences of weights at successive layers are generated i.i.d., (6) corresponds to the Kesten process [Kesten, 1973]. When t→∞, the Kesten process may either converge to a limiting distribution or diverge. In our case, the width of the distribution of entries of $W^{\left(t\right)}$ acts as a parameter that controls the transition between these two qualitatively distinct behaviors.

**Figure 1:**
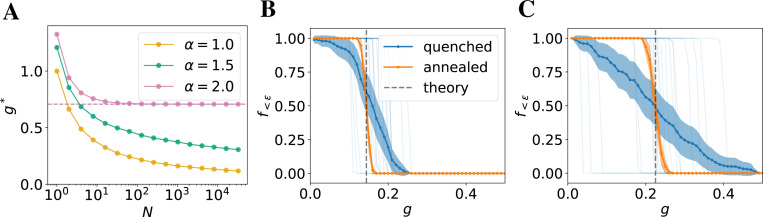
(**A**): Transition point $g^{*}$ predicted by our theory as a function of network size for various α. The transition point of Gaussian networks rapidly converges to the mean-field limit (dashed line). In contrast, the transition point of heavy-tailed networks decays slowly towards zero. (**B**): The fraction of small (ϵ=0.1) final state components in linear networks with α=1 and N=3000 evolved for T=100 steps from random initial conditions as a function of g. In the annealed case, we observe a sharp transition at the location predicted by the theory. In the quenched case, each individual realization exhibits a sharp transition (thin blue lines), but its location varies between different realizations of the weight matrix. Thus, when averaged over the realizations (thick blue line and dots; shaded region shows the ±3 standard error), the transition looks smoother than in the annealed case. Nonetheless, its location is approximately predicted by the theory. (**C**): Same as B but with N=100. As predicted by the theory, the transition point shifts to the right with decreasing N. Moreover, the location of the transition in the quenched case varies more in smaller networks.

A detailed analysis in Appendix A shows that the critical width of the distribution is given by

(7a) $$g^{*}=\exp\left(-\langle {\text{Ξ}}_{N,\alpha }\rangle \right)$$

(7b) $${\text{Ξ}}_{N,\alpha }=\frac{1}{\alpha }\ln\left(\frac{1}{N}\sum_{j=1}^{N}{\left|z_{j}\right|}^{\alpha }\right)$$

with $z_{j}\sim L_{\alpha }(1)$. Let us first show that this formula is consistent with the known results in the Gaussian case. Noting that in our notation for α=2 we have $\langle z^{2}\rangle =2$, we can take the limit N→∞ and obtain ${\text{Ξ}}_{N\to \infty ,2}=\ln\sqrt{2}$. This leads to $g^{*}=1/\sqrt{2}$ and $L_{2}\left(g^{*}\right)\sim N(0,1)$. Hence, we recover the well-known transition at $\langle W_{ij}^{2}\rangle =1/N$ [Sompolinsky et al., 1988, Molgedey et al., 1992, Toyoizumi and Abbott, 2011]. Our formula, however, is more general and applies to any finite width of the network. It predicts that $g^{*}$, for any fixed α, is a decreasing function of N (Fig. 1A). In the Gaussian case, it quickly reaches its asymptotic value consistent with the mean-field prediction. In heavy-tailed networks, however, the decay is slow and is clearly visible across four orders of magnitude shown in Fig. 1A. Our theory predicts that this decay is logarithmic with an α-dependent exponent, i.e., $g^{*}\propto 1/{\left(\ln N\right)}^{1/\alpha }$ for α<2, see Appendix B for the derivation.

We also confirm our theoretical predictions in simulations, by passing a random initial vector through T=100 steps (layers) of a linearized network with weights redrawn at each step from a fixed distribution. We fix α=1 and vary g. Below (above) the transition, we expect the components of the final state to be close to (far from) zero with high probability. Thus, we construct a simple order parameter $f_{<\epsilon }$ defined as the number of components of the final state ε(T) that are within ϵ from 0. As shown in Fig. 1B, the network goes through a sharp transition between $f_{<\epsilon }=1$ and $f_{<\epsilon }=0$, and the location of the transition is consistent with our theoretical prediction. The transition is rather sharp even for small networks (Fig. 1C, N=100), and is expected to become even sharper with increasing number of steps T. Our theoretical result becomes exact in the limit of T→∞.

#### 4.1.2 Quenched disorder and recurrent neural networks

In contrast to our annealed analysis of feedforward networks, the weights of the RNN remain constant throughout the evolution. Since we are interested in finite-sized networks, we can expect the location of the transition to vary between the realizations of the weight matrix. A mathematical analysis of this phenomenon is beyond the scope of this work. We expect, however, that the random fluctuations of $g^{*}$ in networks with quenched random weights should be concentrated around the annealed prediction and should decrease with N. Moreover, the typical values of $g^{*}$ should decrease with N as predicted by the annealed theory.

To test this hypothesis, we simulate a quenched version of (6) in which $\forall_{t}W^{\left(t\right)}=W$. In order to observe the transition, we first fix the random seed (i.e., draw random components of the weight matrix from $L_{\alpha }\left(1/N^{1/\alpha }\right)$) and then rescale its components by various values of g. We focus on α=1 as a representative example. As shown in Fig. 1B, evolution of each realization of the weight matrix goes through a very sharp transition, but the location of this transition varies significantly between the realizations. Nonetheless, they are concentrated around the point predicted by the annealed theory. Moreover, the location of the transition shifts to the right and fluctuations increase with decreasing N and (Fig. 1C). These results suggest that the location of the transition in the quenched case approaches the annealed prediction with increasing N. We provide further analysis on the behavior of the quanched transition point as a function of network size N in Appendix J.

**Figure 2:**
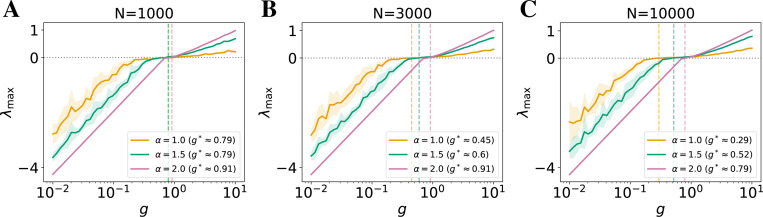
Maximum Lyapunov exponent ($\lambda_{\max}$) as a function of gain g for autonomous recurrent networks with different tail indices α, shown for: (**A**) N=1000, (**B**) N=3000, and (**C**) N=10000. Curves show mean across 10 trials; shaded regions denote ±1 SD. We let the networks evolve for T=3000 steps, among which the Lyapunov exponents are accumulated over the last K=100 steps. See results under noisy stimulus and ablation studies in Appendices E, G. Heavier-tailed networks (lower α) exhibit a slower, more gradual increase in $\lambda_{\max}$ near the transition (where $\lambda_{\max}=0$), resulting in a broader edge-of-chaos regime with respect to g. Dashed lines and legend mark the average critical gain g* at which $\lambda_{\max}$ first crosses zero. As N increases, this transition shifts leftward, especially for lower α, in line with our theoretical predictions on finite-size effects.

Note that our theoretical analysis does not specify the nature of the dynamics above the transition. Our simulations indicate that the network hovers around the edge of chaos in a significant range of values of g and, for α≥1, ultimately enters chaotic regime (see Fig. 2). In contrast, the dynamics of networks with α<1 show a non-monotonic behavior of the MLE: after staying near the edge of chaos at intermediate values of g, the dynamics seem to ultimately settle in a stable, non-chaotic regime at larger values of g (see Appendix D). Thus, for α≥1, our annealed prediction $g^{*}$ gives the approximate location of the transition to chaos in RNNs. Although our analysis focused on autonomous dynamics, similar to the Gaussian case, we expect noise to shift, but not completely remove, the transition [Molgedey et al., 1992, Rajan et al., 2010].

While our analysis focuses on the tanh activation, it generalizes to any function satisfying ϕ(0)=0 and admitting a local expansion ϕ(x)=ax+o(x). In this regime, the existence of the transition follows directly from the linear stability of the quiescent fixed point. For unbounded activations such as ReLU, however, bounded dynamics are no longer guaranteed, and divergence may occur at large g. Although the transition itself persists for a broad class of activations, the qualitative behavior above it can differ substantially. Beyond the transition, the ensuing dynamics depend sensitively on the nonlinearity: linear or ReLU activations typically diverge for large g, whereas sublinear, saturating nonlinearities constrain activity and preserve stability. We therefore expect our results to hold for any smooth, saturating activation function, while unbounded ones likely produce more complex, divergent dynamics. The framework can also be extended to cases with ϕ(0)≠0 by expanding around the corresponding non-quiescent fixed point. Here, the fixed point’s location may vary with the order parameter g, but the overall nature of the transition should remain unchanged.

### 4.2 Heavier-tailed RNNs exhibit a slower, more robust transition to chaos

Having established the existence of a finite-size transition between quiescent and chaotic dynamics in RNNs with heavy-tailed synaptic weights (Section 4.1), we next examine how the nature of this transition differs across tail indices α. Our simulations of autonomous RNNs (Fig. 2; similar results for noisy stimulus-driven RNNs shown in Fig. 5) reveal that although networks with α≥1 exhibit a transition to chaos as predicted, the sharpness and location of the transition vary substantially with α, in which a lower value corresponds to a heavier-tailed distribution.

In networks with Gaussian connectivity (α=2.0), the maximal Lyapunov exponent (MLE) increases steeply with gain g, indicating a rapid onset of chaos. In contrast, RNNs with heavier-tailed weights (lower α) exhibit a slower rise in the MLE as g increases near the transition (when the MLE is near zero). This gradual transition implies that these networks remain closer to the edge of chaos over a wider range of gain values, consistent with previous observations of an extended, critical-like region [Wardak and Gong, 2022, Qu et al., 2022]. Such extended critical-like behavior can offer a form of robustness with respect to changes in network parameters, which can be an important property that benefits biological networks in non-stationary environments, allowing the network to maintain sensitive high-capacity dynamics [Bertschinger et al., 2004, Legenstein and Maass, 2007, Toyoizumi and Abbott, 2011] without the requirement of precise parameter adjustment. In our analysis of reservoir-computing networks on a delayed XOR task (Appendix L), heavy-tailed networks maintained strong task performance across a broader gain regime than Gaussian networks. This provides a concrete proof-of-concept that the extended critical regime enhances robustness and performance without fine-tuning, which may benefit both machine learning applications and neural computation. Future studies could extend this analysis to trained recurrent networks and more complex temporal tasks to further elucidate how heavy-tailed connectivity shapes information processing and learning dynamics.

Moreover, as shown in Fig. 2 (with N increases from left to right panels), the locations of the transition (MLE ≥ 0) shift to the left (lower g) as N increases. Notably, this shift is more pronounced in heavier-tailed networks. This finding echoes our theoretical prediction that the critical gain $g^{*}$ slowly decreases with increasing N in the heavy-tailed regime due to the finite-size effect (Fig. 1A).

Together, these simulation results provide empirical evidence that while infinite-width mean-field theory predicts ubiquitous chaos for Lévy RNNs, finite-size networks can operate near a well-defined, robust transition point whose properties depend systematically on the tail index α and network size N. This behavior may be particularly relevant in biological systems, where recent experimental evidence suggests synaptic weights follow heavy-tailed statistics, and where robustness to parameter variation is essential. Our findings imply that heavy-tailed connectivity may naturally support computations at the edge of chaos in finite-size neural circuits without requiring fine-tuning.

### 4.3 Heavy-tailed RNNs compress the chaotic attractor into a lower-dimensional slow manifold

The robustness of transition to chaos in heavy-tailed RNNs raises a natural question: does the structure of the underlying dynamical landscape also vary systematically with respect to the tail index α?

To address this, we first examine the full Lyapunov spectrum of networks near the transition to chaos, then we further characterize the effective dimensionality of the network’s dynamics using two complementary metrics: the Lyapunov dimension ($D_{\text{KY}}$), which estimates how many directions in phase space are locally expanding or marginally stable [Frederickson et al., 1983, Farmer et al., 1983, Ott, 2002], and the participation ratio (PR), which captures how variance is distributed across neural population activity and is commonly used in neuroscience [Kramer and MacKinnon, 1993, Gao et al., 2017, Recanatesi et al., 2022]. We find that although heavy-tailed networks benefit from robustness near the edge of chaos, this comes with a key tradeoff: the dynamics are compressed into a lower-dimensional slow manifold.

#### 4.3.1 Lyapunov spectrum shows compressed slow manifold in heavy-tailed RNNs

To probe the structure of the dynamical landscape near the transition to chaos, we examine the full Lyapunov spectrum of the networks. The spectrum provides a detailed view of local stability across phase space, with each Lyapunov exponent characterizing the growth or decay of perturbations along a particular direction in the network’s state space. In particular, the density of the exponents near zero reflects the presence of a slow activity manifold where the network evolves in the steady state. The slow manifold contains marginally stable modes along which input-driven perturbations expand or shrink slowly. Thus, in the absence of other memory mechanisms, slow modes endow RNNs with a crucial capacity to integrate information across long timescales [Krishnamurthy et al., 2022].

**Figure 3:**
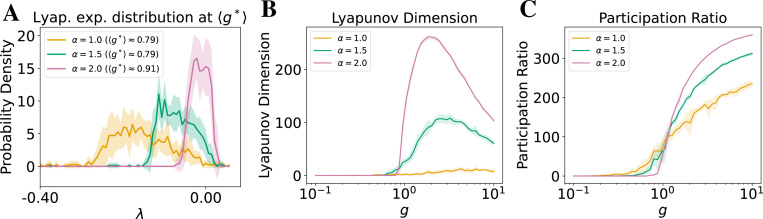
Heavy-tailed networks exhibit lower-dimensional attractors near the edge of chaos. Curves show mean across 10 trials for networks of size N=1000; shaded regions denote ±1 SD. See Appendix E for results under noisy stimuli. The implementation details and ablation studies are provided in Appendices H, I. (**A**) Distributions of top 100 Lyapunov exponents for varying α show fewer exponents near zero in heavier-tailed networks at estimated $\langle g^{*}\rangle$ obtained in Fig 2A, indicating a lower-dimensional slow manifold. x-axis truncated at the left to omit near-zero tails for clarity. (**B**) The Lyapunov dimension is smaller for heavier-tailed networks near the regime of edge of chaos, reflecting fewer directions of local expansion in phase space. (**C**) The participation ratio dimension is similarly smaller with lower α near the edge of chaos, showing reduced variance homogeneity across neural modes. Together, these results indicate that while heavy-tailed networks maintain robustness to neural gain near chaos, they compress dynamics into a lower-dimensional attractor.

In Fig. 3A, we show the Lyapunov spectra for Gaussian and heavy-tailed networks near their respective estimated critical gain when the MLE first exceeds zero. The average critical gain $\langle g^{*}\rangle$ is estimated through ten realizations in Fig. 2A and the histograms are averaged across runs with the same $\langle g^{*}\rangle$, hence they contain positive Lyapunov exponents (see Appendix F for individual realizations across input conditions, and additional discussion on the overestimation of $\langle g^{*}\rangle$). The distribution of Lyapunov exponents differs markedly between these two types of network connectivity. Gaussian networks show a dense band of exponents concentrated near zero, indicating a broad, slow manifold. In contrast, as α decreases (*i.e.,* the heaviness of the tail increases), the number of Lyapunov exponents near zero decreases, revealing a *compression* of the slow manifold.

This suggests a tradeoff between the robustness of the edge of chaos and the dimensionality of the slow manifold. Following this observation, we next quantitatively characterize the attractor dimensionality.

#### 4.3.2 Lyapunov dimensions and participation ratio further characterize low attractor dimensionality in heavy-tailed RNNs

To further characterize the tradeoff introduced by heavy-tailed connectivity, we quantify the dimensionality of the dynamical attractor using two complementary metrics (detailed in Section 3.4).

First, we compute the Lyapunov dimension ($D_{\text{KY}}$), which estimates the effective number of directions in phase space that exhibit local expansion [Frederickson et al., 1983, Farmer et al., 1983, Ott, 2002]. This measure reflects the intrinsic complexity of the system’s attractor. As shown in Fig. 3B, recurrent networks with heavier-tailed synaptic weights (lower α) exhibit a significantly lower $D_{\text{KY}}$ than their Gaussian counterparts across the near-chaotic regime (characterized in Fig. 2). This confirms that despite their robustness to chaos, heavy-tailed networks operate on lower-dimensional attractors.

Second, we evaluate the participation ratio (PR), a widely used metric for gauging the effective dimensionality of neural population activity. It has been leveraged to quantify task-relevant low-dimensional subspaces and other properties of multi-unit neuronal recordings in behaving animals [Gao et al., 2017], and summarize the collective modes visited by recurrent spiking networks and reveal how these modes depend on local connectivity motifs [Recanatesi et al., 2019]. PR measures how variance in population activity is distributed across the eigenmodes of the covariance matrix, providing a compact read-out of the number of degrees of freedom the network explores [Kramer and MacKinnon, 1993]. As shown in Fig. 3C, PR declines as α decreases, although the drop is shallower than that of $D_{\text{KY}}$. This difference is expected: PR is a second-order statistic that is sensitive to how variance is spread across modes, whereas $D_{\text{KY}}$ is a quantity set by local expansion rates of the flow. Consequently, PR can remain relatively high even when only a few directions in phase space are truly unstable, highlighting complementary information provided by these two dimensionality measures.

We hypothesize that the large disparity in Lyapunov dimensions between Gaussian and heavy-tailed networks arises from the broader dispersion of Lyapunov exponents in the latter as shown in Fig. 3A. Intuitively, only a small subset of leading exponents becomes positive near the edge of chaos in heavy-tailed networks, resulting in a lower overall Lyapunov dimension. This effect likely reflects the more heterogeneous eigenvalue distribution of the underlying weight matrix. However, the precise mapping between the weight matrix spectrum and the Jacobian’s Lyapunov spectrum remains nontrivial and warrants further analysis.

Together, these metrics reveal that the slow manifold in heavy-tailed RNNs is both more contractive (lower $D_{\text{KY}}$) and narrower (lower PR), supporting the view that these networks “prioritize” robustness over dynamical richness. This tradeoff is biologically aligned with observations in animal studies, where low-dimensional neural representations are often found relative to the high-dimensional ambient space of neural recordings, even in complex behaviors [Nieh et al., 2021, Cueva et al., 2020, Chaudhuri et al., 2019, Yoon et al., 2013]. We return to this point in the Discussion.

## 5 Discussion

Critically, our findings are with respect to finite-size networks and depend on network size. In the infinite-width limit, mean-field theory predicts that Lévy networks are always chaotic (Section 4.1.1). However, our results show that finite-size networks exhibit a clear quiescent-to-chaotic transition, with the critical gain $g^{*}$ shifting systematically with both network size N and tail index α (Eqn. 7 and Fig. 2). This highlights that mean-field approximations may miss important structure in biologically sized circuits, and that finite-size corrections offer a more accurate theoretical framework for understanding real neural systems that are finite in size.

Further, as demonstrated in Fig. 2, heavy-tailed weight distributions make RNNs more robust to changes in gain, a parameter that may correspond biologically to either the width of synaptic weight distributions or to neural gain modulated by neuromodulatory systems [Waterhouse et al., 1988, Shine et al., 2018]. Specifically, we observe that networks with heavier-tailed synaptic weights remain near the edge of chaos over a much wider range of gain values than those with Gaussian connectivity, which is commonly assumed in theoretical studies. This property may be especially valuable for biological systems that operate across multiple states (e.g., sleep and waking [Chaudhuri et al., 2019]) or in non-stationary environments. Such robustness could help explain empirical findings that similar neural activity patterns can arise from vastly different underlying circuit parameters in healthy brains [Prinz et al., 2004, Marder, 2011]. Meanwhile, as shown in Fig. 3, heavier tails reduce both the Lyapunov dimension and the participation ratio, indicating that the slow manifold supporting long-lasting activity becomes lower-dimensional. Our further analysis show that a handful of “mega-synapses” drives the dynamics, implying the robustness and low-dimensionality largely stem from extreme outliers (Appendix K). Together, these effects imply a tradeoff: heavy-tailed networks are more stable to perturbations but require more neurons to achieve the same computational capacity, such as memory or temporal integration, compared to Gaussian networks.

Notably, a common empirical observation in neuroscience is that neural population activity tends to evolve within a low-dimensional manifold relative to the large number of neurons recorded. This phenomenon has been observed across cortical and subcortical regions, and is often behaviorally meaningful [Nieh et al., 2021, Bondanelli et al., 2021]. Theoretical work suggests that low-dimensionality can arise from constraints imposed by circuit connectivity [Mastrogiuseppe and Ostojic, 2018] or task demands [Gao et al., 2017]. Our finding that heavier-tailed RNNs yield lower-dimensional attractors biologically aligns with this widespread phenomenon and provides evidence that anatomical connectivity might constrain the expressive capacity of population activity.

The observed robustness-dimensionality tradeoff also offers predictions for which tasks heavy-tailed circuits can be best suited. Tasks requiring only low-rank dynamics for reliable integration or pattern generation (*e.g.,* binary decision-making [Brunton et al., 2013] or working memory [Panichello and Buschman, 2021]) may benefit from the extended edge-of-chaos regime provided by heavy-tailed weights. In contrast, tasks that rely on high-dimensional dynamics—such as representing multiple independent memories or generating complex trajectories (*e.g.,* virtual reality navigation [Busch et al., 2024])—may require larger networks or connectivity distributions closer to Gaussian. These predictions can be tested using emerging connectomic [Dorkenwald et al., 2024, The MICrONS Consortium, 2025] and large-scale recording datasets [Bondy et al., 2024, Manley et al., 2024], which can jointly measure synaptic weight distributions and task-related activity dimensionality.

Our framework can be naturally extended to a mixture setting, improving biological plausibility. For instance, neurons could be homogeneous and each draw weights randomly from one of multiple heavy-tailed distributions or form interacting subpopulations with distinct α values and connectivity motifs. Such extensions may capture diversity across neuronal cell types [Jin et al., 2025, Zeng, 2022] and offer a promising direction for future work.

We acknowledge several limitations: our study used untrained rate-based networks with homogeneous units. Including more biologically realistic features such as spiking dynamics [Kim et al., 2019], Dale’s law [Dale, 1935], cell-type diversity [Zeng, 2022, Yao et al., 2023], and synaptic plasticity [Citri and Malenka, 2008] could modify or refine the observed effects. Furthermore, while our results focused on untrained dynamics, a key next step is to study how learning algorithms interact with the broad critical regime and how trained or reservoir computing heavy-tailed networks perform across a range of tasks [Yang et al., 2019, Driscoll et al., 2024]. Such a study would help us to understand and predict, based on connectivity alone, what kinds of computations a brain-like circuit is suited to perform—an important goal as we seek to interpret rich new connectomic datasets and understand how synaptic connectivity ties to function [Garner et al., 2024, Seung, 2024]. Another valuable next step is to extend this work toward direct comparison with neural recordings. For example, future studies could estimate Lyapunov spectra or related dynamical signatures from long, high-resolution neural activity trajectories. While such analyses are technically challenging and require stable, extended recordings, they would offer a powerful bridge between theory and experiment.

In summary, finite-size recurrent networks with previously understudied Lévy-distributed weights reveal a clear rule: heavier-tailed synaptic connectivity widens the regime of stable, edge-of-chaos dynamics but reduces the dimensionality of the resulting activity. This tradeoff links connectivity statistics, network size, and functional capacity, offering a principled, biologically plausible framework for interpreting both biological data and designing more parameter-robust artificial systems.

## Supplementary Material

Supplement 1
