# Supplementary material for: Slow Transition to Low-Dimensional Chaos in Heavy-Tailed Recurrent Neural Networks
Source: ArXiv. 2025 Oct 24:arXiv:2505.09816v2. Preprint. [Version 2] (PMC12633624)
Supplement: Supplement 1 [file NIHPP2505.09816v2-supplement-1.pdf]

A Mathematical analysis of the transition in annealed networks

Our goal is to show that networks with α-stable weight distributions exhibit a transition between two regimes, and to find the location of this transition which we denote as g^*. As in Gaussian networks, the quiescent state is stable and any small perturbation around it shrinks if weights are generated from a narrow enough distribution (i.e., $g < g^*$). Similarly, the quiescent state is unstable if the underlying distribution is wide enough ($g > g^*$). In contrast to Gaussian networks, however, this effect can only be observed through the analysis of finite-size effects.

As described in the main text, we study linear stability of the quiescent fixed point of (6). Since weights are randomly redrawn at each step, the evolution $\varepsilon^{(t)}$ is a stochastic process. To quantify its behavior we focus our attention on the conditional distribution $\varepsilon^{(t+1)}$ given $\varepsilon^{(t)}$. Components of this vector are independent due to the assumed independence of rows of the weight matrix. The conditional distribution of a single component can be characterized in the Fourier space as

$$\left\langle \exp\left(ik\varepsilon_i^{(t+1)}|\varepsilon^{(t)}\right)\right\rangle_W = \left\langle \exp\left(ik\sum_{j=1}^{N_t} W_{ij}^{(t)}\varepsilon_j^{(t)}\right)\right\rangle_{W_{ij}^{(t)}} = \exp\left(-|k|^\alpha g^\alpha \frac{1}{N_l}\sum_{j=1}^{N_t}\left|\varepsilon_j^{(t)}\right|^\alpha\right) \tag{8}$$

where we used $W_{ij}^{(t)} \sim L_\alpha\left(g/N_t^{1/\alpha}\right)$. Thus, for $t > 1$ the perturbation, when conditioned on the previous step, is an α-stable random variable. More specifically, it can be written as $\varepsilon_i^{(t+1)}|\varepsilon^{(t)} \sim L_\alpha\left(\gamma^{(t+1)}\right)$, where the conditional scale at step $t+1$

$$\gamma^{(t+1)} = g\left(\frac{1}{N_t}\sum_{j=1}^{N_t}\left|\varepsilon_j^{(t)}\right|^\alpha\right)^{1/\alpha} \tag{9}$$

is a deterministic function of state at time t, which itself is a random variable. We can unpack this relation one step backwards by conditioning on $\varepsilon^{(t-1)}$ instead, with $\varepsilon_i^{(t)}|\varepsilon^{(t-1)} \sim L_\alpha\left(\gamma^{(t)}\right)$. We utilize the fact that this can also be expressed as

$$\varepsilon_i^{(t)}|\varepsilon^{(t-1)} = \gamma^{(t)} z_i^{(t)} \tag{10}$$

where $\gamma^{(t)}$ depends on the perturbation at time $t-1$, and $z_i^{(t)}$ are i.i.d. α-stable variables. This leads to the recursive formula for scalar $\gamma^{(t)}$

$$\gamma^{(t+1)} = \gamma^{(t)}\xi^{(t)} \tag{11}$$

where $\left(\xi^{(t)}\right)_{t=1}^\infty$ is a sequence of independent random variables distributed as

$$\xi^{(t)} = g\left(\frac{1}{N_t}\sum_{j=1}^{N_t}\left|z_j^{(t)}\right|^\alpha\right)^{1/\alpha} \tag{12}$$

with i.i.d. $z_j^{(l)} \sim L_\alpha(1)$. If layers have the same width $N_t = N$, $\xi^{(t)}$ are i.i.d. and (11) is a scalar multiplicative process with i.i.d. entries. Thus, we have reduced our problem to a simpler special case of purely multiplicative scalar Kesten process. We can easily solve this recursion and rewrite the solution as a sum

$$\ln\gamma^{(t+1)} = \ln\gamma^{(t)} + \sum_{i=1}^{t}\ln\xi^{(i)} \tag{13}$$

where $\gamma^{(1)}$ is deterministically specified by the input perturbation $\varepsilon^{(0)}$. It is known [Kesten, 1973, Statman et al., 2014] that this sum diverges to $-\infty$ almost surely if $\langle\ln\xi\rangle < 0$ and diverges to ∞ almost surely if $\langle\ln\xi\rangle > 0$. Accordingly, the sequence $\left(\gamma^{(t)}\right)_{t=1}^\infty$ either converges to 0 or diverges. Therefore, the critical width of the synaptic weight distribution is given by

$$g^* = \exp\left(-\langle\Xi_{N,\alpha}\rangle\right) \tag{14}$$

where

$$\Xi_{N,\alpha} = \frac{1}{\alpha}\ln\left(\frac{1}{N}\sum_{j=1}^{N}|z_j|^\alpha\right) \tag{15}$$

with $z_j \sim L_\alpha(1)$.

B Derivation of the logarithmic decay of $g^*(N)$

Here, we estimate the expected value of

$$\Xi_{N,\alpha} = \frac{1}{\alpha} \ln \left(\frac{1}{N} \sum_{j=1}^{N} |z_j|^\alpha \right), \tag{16}$$

where $z_j \sim L_\alpha(1)$, for large N. We define $Y_{N,\alpha} = \frac{1}{N} \sum_{j=1}^{N} |z_j|^\alpha$ and note that the Laplace transform of $Y_{N,\alpha}$ can be calculated as

$$F_{N,\alpha}(s) = \langle e^{-sY_{N,\alpha}} \rangle = \left(F_{1,\alpha} \left(\frac{s}{N} \right) \right)^N \tag{17}$$

where

$$F_{1,\alpha}(s) = \left\langle e^{-s|z|^\alpha} \right\rangle_{z \sim L_\alpha(1)} \tag{18}$$

According to (17), the large N asymptotic of $\Xi_{N,\alpha}$ is dominated by the behavior of $F_{1,\alpha}(s)$ around $s = 0$. This behavior should be similar for all symmetric distributions with the same stability index. For example, take $\rho_z(x) = \frac{\alpha}{2}|x|^{-1-\alpha}$ for $|x| > 1$ and $\rho_z(x) = 0$ otherwise. The resulting expansion can be found as

$$\left\langle e^{-s|x|^\alpha} \right\rangle_{z \sim \rho_z} = s \int_s^\infty du u^{-2} e^{-u} = s\Gamma(-1, s) \approx 1 - s\,(1 - \gamma - \ln s) + O(s^2) \tag{19}$$

where $\Gamma(a, s)$ is the upper incomplete gamma function and γ is the Euler-Mascheroni constant. Thus, the asymptotic expansion of $F_{1,\alpha}(s)$ must take the form

$$F_{1,\alpha}(s) = 1 - A_\alpha s\,(B_\alpha - \ln s) + O(s^2) \tag{20}$$

for some irrelevant constants A_α, B_α. We plug (20) into (17) and arrive at

$$\ln F_{N,\alpha}(s) = -A_\alpha s\,(B_\alpha - \ln s + \ln N) + O\left(N^{-1}\right) \tag{21}$$

For $N \gg 1$, (21) corresponds to a random variable X_N that can be constructed as

$$X_N = X_1 + A_\alpha \ln N, \tag{22}$$

where

$$\langle \exp(-sX_1) \rangle = \exp(-A_\alpha s\,(B_\alpha - \ln s)) \tag{23}$$

We can rewrite the desired expected value as

$$\langle \Xi_{N,\alpha} \rangle \approx \frac{1}{\alpha} \langle \ln (X_1 + A_\alpha \ln N) \rangle_{X_1} \tag{24}$$

The distribution of X_1 is fixed and does not change with N. Thus, for large N the second term dominates, and we arrive at

$$g^* = \exp(-\langle \Xi_{N,\alpha} \rangle) \asymp \frac{1}{(\ln N)^{1/\alpha}} \tag{25}$$

C Algorithm to compute Lyapunov exponents for RNNs

We leverage the algorithm proposed in Vogt et al. [2022] to study the dynamics of RNNs, adapted to our setting where we process a single input sequence at a time (*i.e.*, batch size = 1). Then, running multiple realizations simply means running the same algorithm but with a different seed set in the beginning; this is equivalent to having a batch of inputs shown in the original algorithm in Vogt et al. [2022].

To reduce the influence of transient dynamics, we include a `warmup` period during which the RNN is evolved forward but Lyapunov exponents are not yet accumulated.

In this procedure, x_t is the input at time step t, h is the hidden state of the RNN, Q is an orthogonal matrix that evolves to track an orthonormal basis in tangent space, $J = \frac{df}{dh}$ is the Jacobian of the RNN dynamics with respect to the hidden state, R is the upper-triangular matrix from the QR decomposition, and γ_i accumulates the log-magnitudes of the diagonal entries R_{ii}. The accumulation begins only after the `warmup` steps, and the final Lyapunov exponent λ_i is computed by normalizing γ_i by the number of post-`warmup` accumulation steps, which is $K = T - \texttt{warmup}$.

Algorithm 1: Lyapunov Exponents Calculation

Initialize h, Q;
for $t = 1$ **to** T **do**
    $h \leftarrow f(h, x_t)$;
    if $t >$ *warmup* **then**
        $J \leftarrow \frac{df}{dh}$;
        $Q \leftarrow J \cdot Q$;
        $Q, R \leftarrow \texttt{qr}(Q)$;
        $\gamma_i \mathrel{+}= \log(R_{ii})$;
$\lambda_i = \gamma_i / (T - \text{warmup})$

D Lack of transition to chaos for $\alpha = 0.5$

A shown in Fig. 4, networks with $\alpha = 0.5$ do not seem to transition to chaos. For small values of g, the MLE increases with g as expected from the stability analysis. Moreover, similarly to other heavy-tailed networks, they hover close to the edge of chaos for a wide range of values of g. However, for larger values of g the MLE starts decreasing with g again and, as a result, usually stays negative for all values of g. This effect seems to persist for noisy inputs (Fig. 5) and other changes in the parameters of the simulations (Figs. 9 and 10). More work is required to explain the source of this interesting phenomenon. However, since in this study we focus our attention on transition to chaos, for clarity we exclude $\alpha < 1$ from most figures.

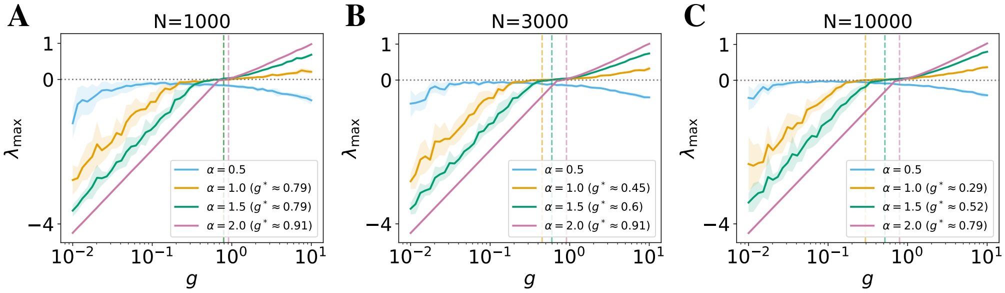

Figure 4: Same as Fig. 2, but with an addition of $\alpha = 0.5$.

E Additional results under small noisy input

We replicate our main results under a small i.i.d. Gaussian noise drive (variance = 0.01) sampled at each time step to test the robustness of the quiescent-to-chaotic transition and attractor geometry in more biologically realistic, stimulus-driven settings. Despite the added input variability, which quenches chaos as expected Molgedey et al. [1992], the trends largely mirror the autonomous case.

Figure 5 shows the maximum Lyapunov exponent (MLE) as a function of gain g across network sizes ($N = 1000, 3000, 10000$) and tail indices α. Heavier-tailed networks ($\alpha < 2$) exhibit a more gradual increase in MLE and an extended edge-of-chaos regime, consistent with Fig. 2. The transition point shifts leftward with increasing N, in line with our mathematical finite-size predictions.

Figure 6 characterizes the attractor dimensionality using Lyapunov spectra, Lyapunov dimension (D_{KY}), and participation ratio (PR). While D_{KY} and the spectrum remain consistent with the autonomous case, PR displays a U-shaped profile (Fig. 6C), unlike the monotonic rise seen in Fig. 3B. This dip likely reflects a shift in the dominant dynamics: at low g, noise drives weak, independent fluctuations across neurons; near the transition, recurrent dynamics compress activity into an elongated low-dimensional manifold; at higher g, chaotic expansion increases PR. Thus, while the robustness-dimensionality tradeoff holds under noisy input, noise modulates how variance is distributed across neural modes.

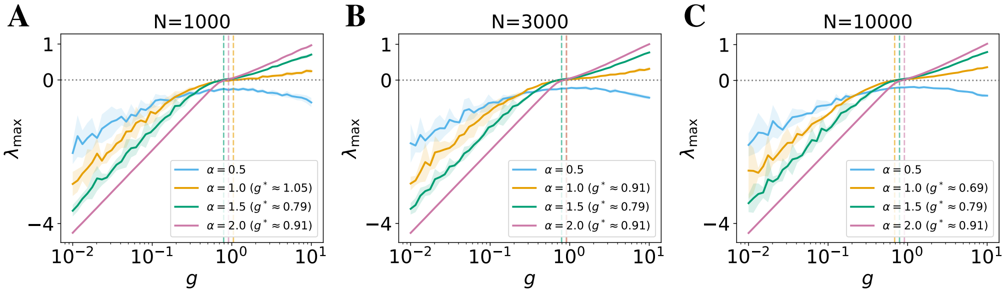

Figure 5: **Effect of network size under small i.i.d. noisy input.** Maximum Lyapunov exponent ($\lambda_{\max}$) as a function of gain g in noisy stimulus-driven recurrent networks with Lévy α-stable weight distributions. Curves show mean across 10 trials; shaded regions denote ± 1 SD. Each panel corresponds to a different network size: (A) $N = 1000$, (B) $N = 3000$, and (C) $N = 10000$. Curves show mean across 3 trials; shaded regions denote ± 1 SD. As in the autonomous case, if a transition exists, then heavier-tailed networks exhibit a slower transition and wider critical regime near $\lambda_{\max} = 0$. The critical gain g^* (dashed line) shifts leftward with increasing N, consistent with finite-size theory.

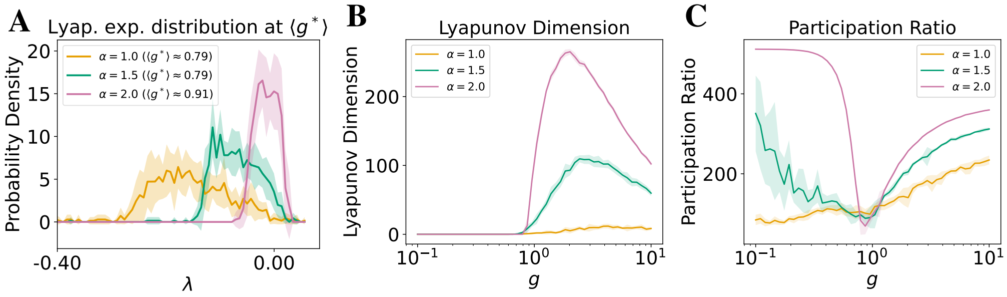

Figure 6: **Attractor geometry under noisy input (N = 1000).** Curves show mean across 10 trials; shaded regions denote ± 1 SD. (A) Lyapunov exponent distributions at the estimated transition point g^*. Heavier-tailed networks exhibit fewer near-zero exponents, indicating a compressed slow manifold. x-axis truncated at the left to omit near-zero tails for clarity. (B) Lyapunov dimension declines with heavier tails, confirming lower attractor dimensionality as found in the autonomous networks. (C) Participation ratio shows a distinct dip near transition to chaos before rising, unlike the monotonic profile observed in the autonomous case, but it is consistently lower in heavier-tailed networks otherwise.

F Visualizations of multiple realizations of Lyapunov spectrum

To assess the variability across realizations of network connectivity, we visualize the full Lyapunov spectrum from three independent trials (different seeds) for networks with $N = 1000$, across both autonomous and noisy stimulus-driven settings. These spectra are computed near the estimated critical gain g^* (obtained in Figs. 2 and 5), where the maximum Lyapunov exponent $\lambda_{\max}$ first crosses zero in each condition. The same value of $\langle g^* \rangle$, computed by averaging g^* of multiple runs, is used across different seeds in these figures. The actual transition point can vary in each realization. Moreover, due to the finite resolution of the grid of g values used in simulations, g^* is overestimated in each seed. Thus, in some realizations, the right edge of the histogram may exceed 0, and the average histograms presented in Figs. 3A and 6A can feature some positive Lyapunov exponents. A more precise estimate could be obtained through a finer-grained or binary search over gain values near the transition point. However, this additional numerical precision would unlikely affect our overall conclusions.

In both autonomous (Fig. 7) and noisy stimulus-driven cases (Fig. 8), Gaussian networks exhibit a dense cluster of exponents near zero, indicative of a broad slow manifold. In contrast, heavier-tailed networks (lower α) show more widely dispersed exponents with fewer near-zero values, consistent with the compression of the slow manifold described in Section 4.3.1. Despite random initialization, the qualitative trend—greater spectrum spread and fewer marginal directions as α decreases—remains consistent across seeds. Notably, in the noisy stimulus-driven case (Fig. 8), the exponents tend to shift downward, and their distributions become more skewed, particularly for heavier-tailed networks. These effects likely reflect interactions between stochastic input and the network's intrinsic dynamics, where the noise quenches the chaos.

Together, these visualizations reinforce our claim that heavy-tailed connectivity leads to systematically lower-dimensional attractors, regardless of input conditions or initializations.

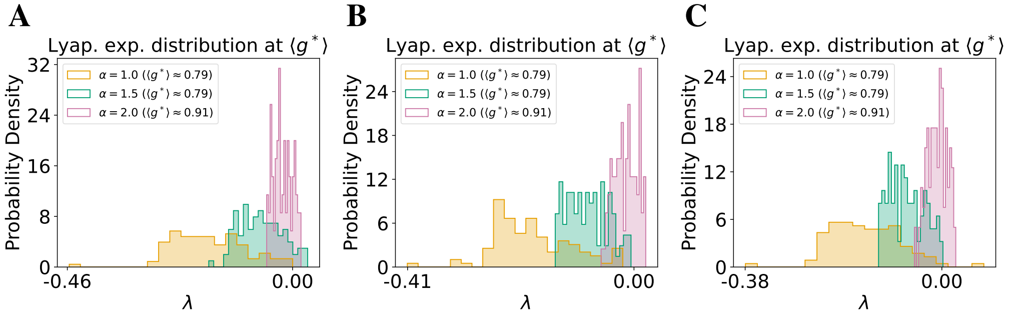

Figure 7: **Full Lyapunov spectra across random initializations in autonomous networks ($\mathbf{N = 1000}$).** Each panel shows the Lyapunov exponent distributions near estimated g^* for an independent seed. Across seeds, heavier-tailed networks (lower α) exhibit a broader spectrum with fewer exponents near zero, indicating reduced slow-manifold dimensionality compared to Gaussian networks.

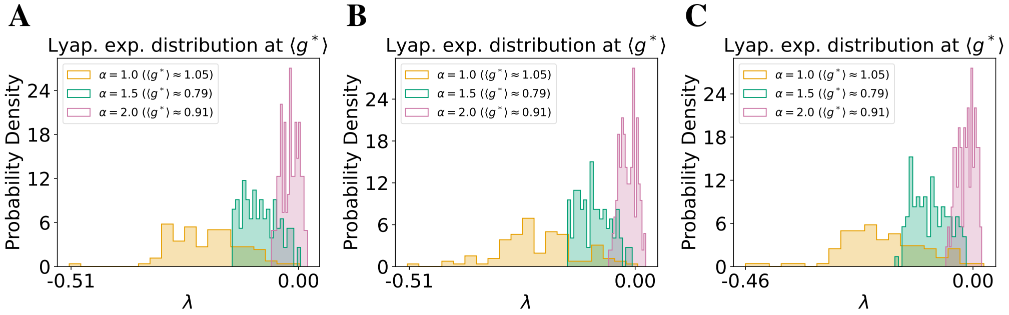

Figure 8: **Full Lyapunov spectra across random initializations in noisy stimulus-driven networks ($\mathbf{N = 1000}$).** Each panel shows the Lyapunov exponent distributions at the estimated critical gain g^* for an independent seed. Spectra under noise remain qualitatively similar to the autonomous case.

G Robustness of results shown in Fig. 2

Since we only examine the maximum (top-1) Lyapunov exponent in Fig. 2, the number of top exponents computed (denoted `k_LE` in the codebase) is irrelevant as long as `k_LE` > 1. Throughout Fig. 2 and this appendix, we use the default `k_LE` $= 100$. Additionally, we exclude an initial warmup period before accumulating exponents to avoid contamination from transients (Appendix C). In Fig. 2, the network is run for $T = 3000$ steps, and Lyapunov exponents are accumulated over the final $K = 100$ steps.

Note that computational cost increases with network size N, number of exponents `k_LE`, accumulation duration K, and total time steps T. Here, we verify that our results in Figs. 2 and 5 are robust to these choices by comparing the default configuration against two more computationally demanding variants, keeping all else fixed:

1. Accumulating exponents over a longer period ($K = T -$ `warmup` $= 150$);
2. Running the network for longer total time ($T = 4000$ with `warmup` of 3900, fixing $K = 100$).

The results are shown in Fig. 9 (autonomous) and Fig. 10 (noisy). All curves remain nearly identical across conditions, demonstrating that our findings are not sensitive to the specific accumulation duration or simulation length. In practice, using $T = 3000$ and $K = 100$ strikes a good balance between computational efficiency and accuracy, especially for large N. These findings validate that the trends reported in Figs. 2 and 5 are robust, and additional compute is not necessary. Note that the effect of network size N has been evaluated in Figs. 2 and 5, in which the critical transition g^* shifts to the left as N increases due to the finite-size effect.

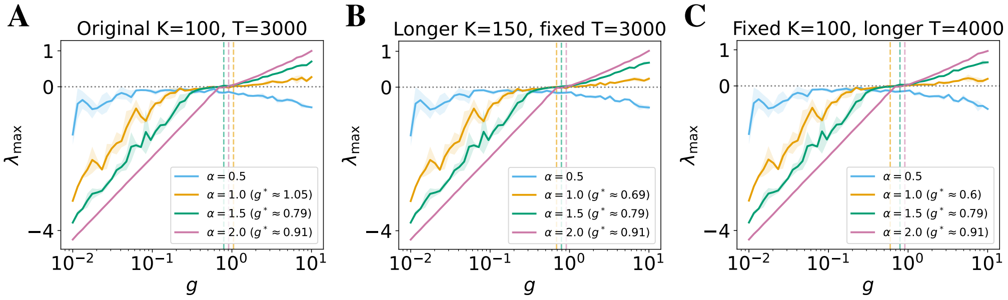

Figure 9: **Robustness of MLE to time horizon and accumulation duration (autonomous, N $=$ 1000).** Curves show mean across 3 trials; shaded regions denote ± 1 SD. (A) Default configuration: $T = 3000$, $K = 100$; (B) Longer accumulation: $K = 150$; (C) Longer sequence: $T = 4000$ with $K = 100$. Results are nearly identical, confirming that the choice of T and K does not affect the reported trends.

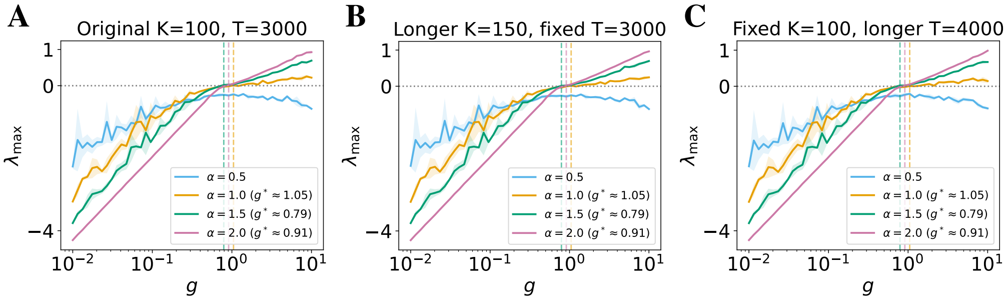

Figure 10: **Robustness of MLE to time horizon and accumulation duration (noisy stimulus-driven, N $=$ 1000).** Curves show mean across 3 trials; shaded regions denote ± 1 SD. (A) Default configuration: $T = 3000$, $K = 100$; (B) Longer accumulation: $K = 150$; (C) Longer sequence: $T = 4000$ with $K = 100$. Results remain stable, indicating that stochastic input does not impact the robustness of MLE computation.

H Robustness of results shown in Fig. 3A

We showed the representative top 100 Lyapunov exponents (`k_LE` = 100) in Fig. 3A using networks of size $N = 1000$. As we are primarily interested in the region near $\lambda = 0$, this choice of `k_LE` is sufficient and larger values do not change the results.

In both Fig. 3A and the visualizations in Appendix F, networks were evolved for $T = 3000$ time steps, with the exponents accumulated over the final $K = 100$ steps, after an initial warmup. As with all our experiments, computation becomes more expensive as the network size N, number of exponents `k_LE`, accumulation duration K, and total time steps T increase. Here we test the robustness of our findings in Fig. 3A by varying these computational parameters, holding all else fixed:

1. Increasing network size to $N = 3000$ (panels A);
2. Accumulating over a longer time window $K = 150$ (panels B);
3. Increasing the total simulation length to $T = 4000$ while maintaining $K = 100$ (using a longer warmup of 3900, panels C).

The resulting spectra, shown below in both autonomous (Fig. 11) and noisy stimulus-driven networks (Fig. 12), are qualitatively the same as the original results. The shape of the Lyapunov spectrum remains consistent: Gaussian networks show a dense band near zero, and heavier-tailed networks exhibit broader spectra with fewer exponents near zero. These results confirm that our main finding—compression of the slow manifold with decreasing α—is robust across a range of network sizes and simulation settings. For large-scale experiments, using $N = 1000$, $T = 3000$, and $K = 100$ provides a reliable and computationally efficient default.

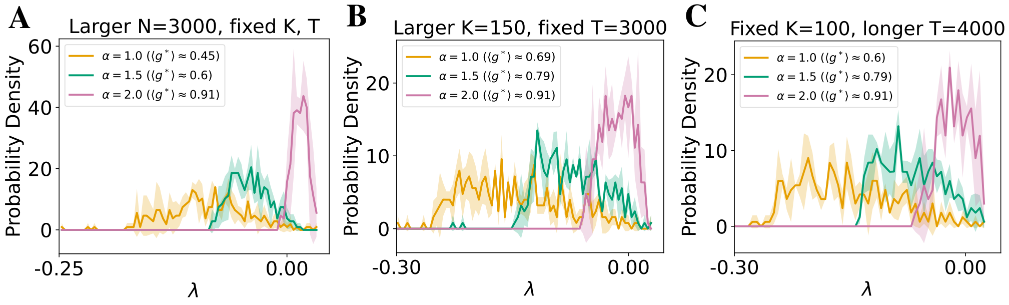

Figure 11: **Robustness of Lyapunov spectra to simulation and accumulation parameters (autonomous).** Curves show mean across 3 trials; shaded regions denote ± 1 SD. Mean Lyapunov spectra near g^* under three conditions: (A) larger network size ($N = 3000$); (B) longer accumulation period ($K = 150$); (C) longer total simulation length ($T = 4000$). Both (B) and (C) use $N = 1000$. The compressed spectrum in heavier-tailed networks remains consistent across all conditions.

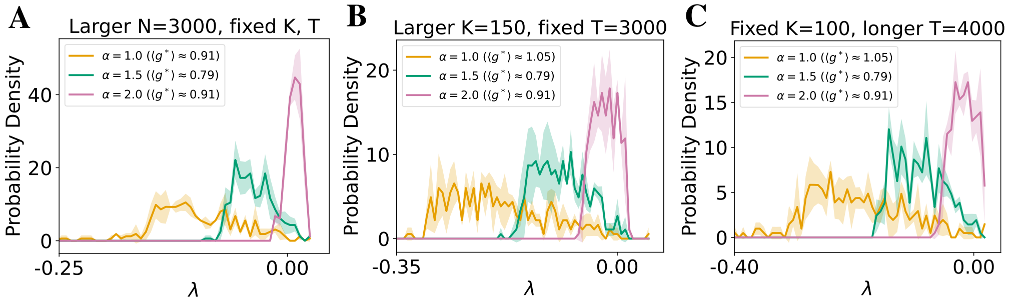

Figure 12: **Robustness of Lyapunov spectra to simulation and accumulation parameters (noisy input).** Same conditions as Fig. 11, but for noisy stimulus-driven networks. Despite stochastic input, heavier-tailed networks continue to exhibit a wider Lyapunov spectrum with fewer marginally stable directions as indicated by the Lyapunov exponents being zero.

I Robustness of results shown in Fig. 3B,C

Lyapunov dimension We use the full Lyapunov spectrum to compute the results shown in Fig. 3B due to the definition of Lyapunov dimension (Eqn. 4), hence k_LE $= N = 1000$ in Fig. 3B. We simulate the dynamics over a total number of $T = 2950$ steps, and use the last $K = 50$ steps to compute the Lyapunov dimension.

Participation ratio To ensure a well-defined participation ratio (PR, Eqn. 5), we require $K > N$, where N is the network size and $K = T -$ warmup denotes the number of time steps used for computing PR after the network has evolved for a number of warmup steps. This condition guarantees that the empirical covariance matrix S, computed from K samples of N-dimensional hidden states, is full-rank and not rank-deficient. When $K \leq N$, S becomes singular or ill-conditioned, leading to unreliable estimates of its eigenvalue spectrum and thus of the participation ratio. In Fig. 3C, we use $T = 2900 + N + 50 = 3950$, meaning 2900 warmup steps with an accumulation period over the last 1050 steps.

Note that the computation cost increases as N, k_LE, K, and T increase.

Here we show our results in Fig. 2 is robust, meaning it is consistent with the more computationally demanding case(s) with all else fixed:

1. Bigger network size $N = 3000$ (panels A);
2. Longer accumulation period $K = 100$ for computing Lyapunov dimension, and longer $K = 1100$ for computing participation ratio (panels B);
3. Longer time trajectory $T = 3950$ for computing Lyapunov dimension and $T = 4950$ for computing participation ratio (panels C).

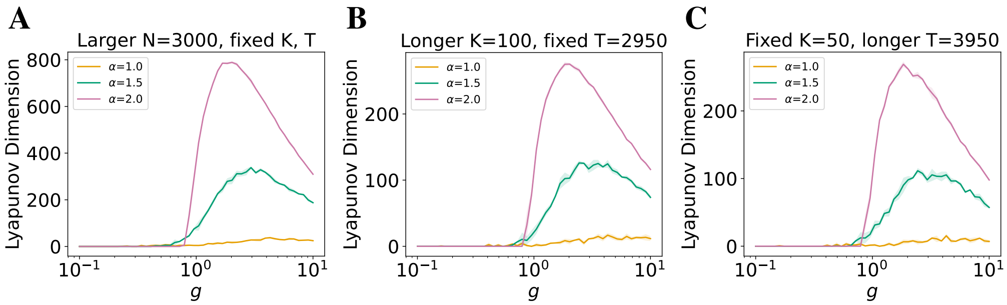

Figure 13: **Robustness of Lyapunov dimension to simulation parameters (autonomous).** (A) Larger network size $N = 3000$; (B) Longer accumulation period $K = 100$; (C) Longer total sequence $T = 3950$ with $K = 50$. All trends remain consistent with those in Fig. 3B.

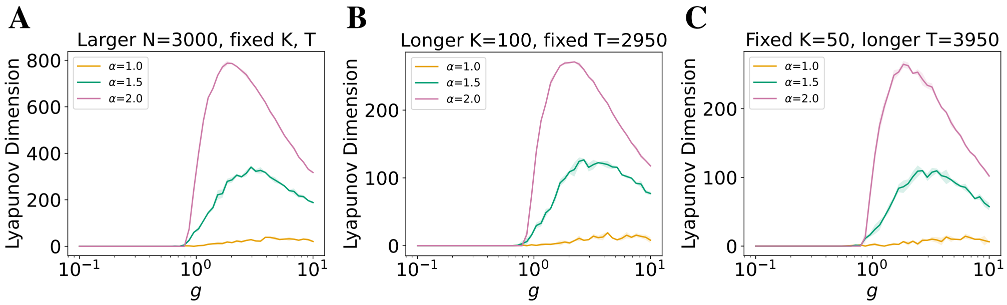

Figure 14: **Robustness of Lyapunov dimension to simulation parameters (noisy).** Same settings as Fig. 13, but with i.i.d. Gaussian input. Results are stable across conditions, confirming robustness of D_{KY} in noisy networks, consistent with those in Fig. 6B.

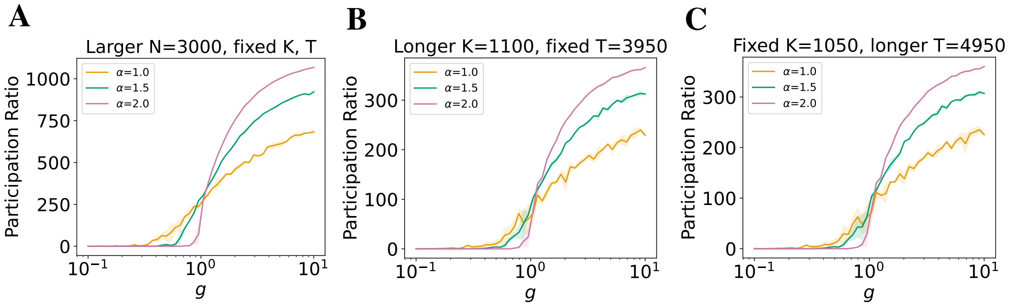

Figure 15: **Robustness of participation ratio to simulation parameters (autonomous).** (A) Larger network size $N = 3000$, $K = 3050$; (B) Longer accumulation period $K = 1100$; (C) Longer sequence $T = 4950$, $K = 1050$. All curves are consistent with Fig. 3C, confirming stability of PR under varying conditions.

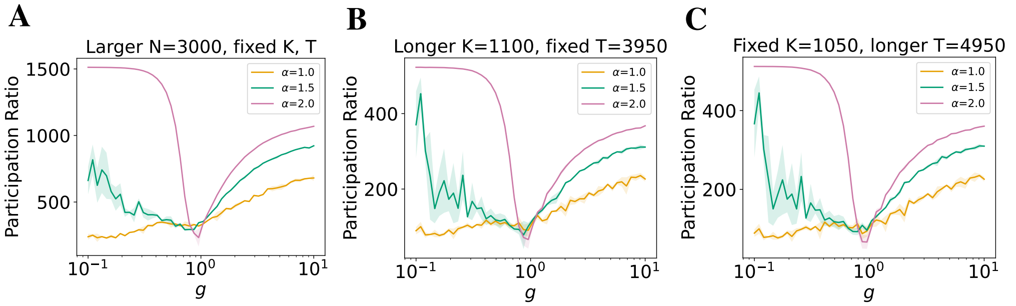

Figure 16: **Robustness of participation ratio to simulation parameters (noisy).** Same configurations as Fig. 15, but under i.i.d. Gaussian input. The non-monotonic profile and overall trends in PR are preserved across all tested conditions, consistent with those in Fig. 6C.

J Behavior of the quenched transition point as a function of N

In finite-sized quenched networks, the location of the transition point fluctuates between realizations of the weight matrix. Since our annealed theory does not offer any insight into the nature of these fluctuations, we resorted to numerical simulations to study how the statistics of g^* scale with N. The results for the representative case of $\alpha = 1$ are shown in Fig. 17. The mean location of the transition point scales like $1/\ln N$, in line with our theoretical predictions (Fig. 17A). The annealed prediction seems to underestimate the true mean over quenched realizations. The standard deviation of g^* decreases with N at a comparable rate as the mean (Fig. 17B). The coefficient of variation of g^* falls off slowly in the studied range of N (Fig. 17C), suggesting that the location of the transition may be (weakly) self-averaging [Wiseman and Domany, 1998] in this system.

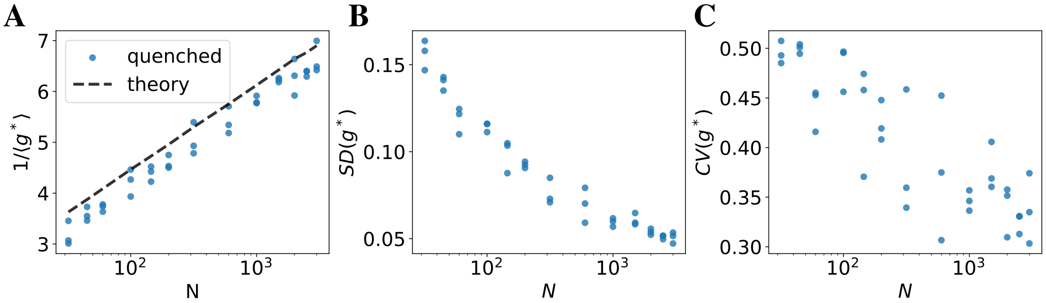

Figure 17: **Statistics of g^* in quenched networks as functions of N.** Note the logarithmic scale on the x-axis. Each point corresponds to the statistics estimated using 100 independent realizations of the weight matrix. For each value of N, we included three data points that correspond to independent estimates calculated based on different random seeds. (A) Reciprocal of the mean. (B) Standard deviation. (C) Coefficient of variation.

K The effect of mega-synapses on dynamics

To test whether robustness and low dimensionality arise from global heavy-tailed statistics or a few extreme “mega-synapses,” we pruned recurrent weights in a network of size $N = 1000$ by absolute magnitude (bottom 95%, top 1%, top 3%) and report the results averaged across three trials. The slow transition vanished when top outliers were removed, shifting the critical gain g^* to larger values, whereas pruning the weakest 95% had little effect (Fig. 18).

Similarly, removing the bottom percentage of weights has very little effect on the general trend of attractor dimensionality. However, when top outliers are removed, the changes are more nuanced: the attractor dimensionality for heavy-tailed weights increases in the chaotic regime, while the transition to chaos is pushed to a larger g^* when more top outlier weights are pruned as mentioned above (Figs. 19, 20). The general ranking of dimensionality by α is largely consistent with the main paper for both dimension measures, though the max dimensionality of $\alpha = 1.5$ is comparable to that of $\alpha = 2$ over a range of g when top outliers are pruned.

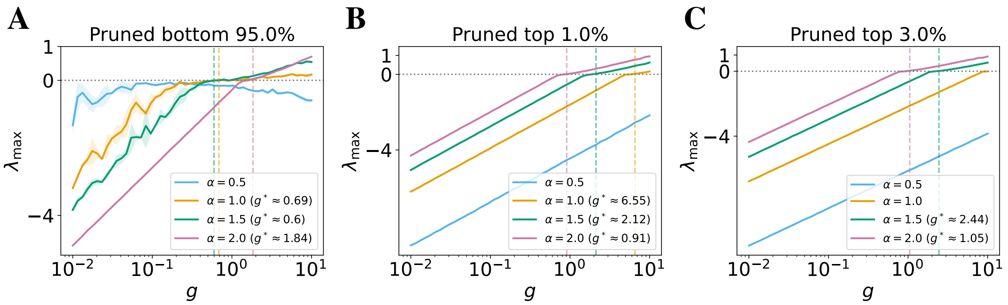

Figure 18: **Effect of pruning on critical gain** g^***.** (A) bottom 95% removed. (B) top 1% removed. (C) top 3% removed.

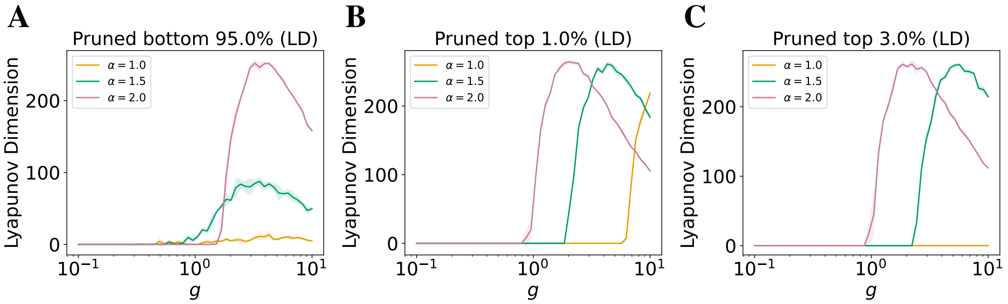

Figure 19: **Effect of pruning on Lyapunov dimension.** (A) bottom 95% removed. (B) top 1% removed. (C) top 3% (LD) removed.

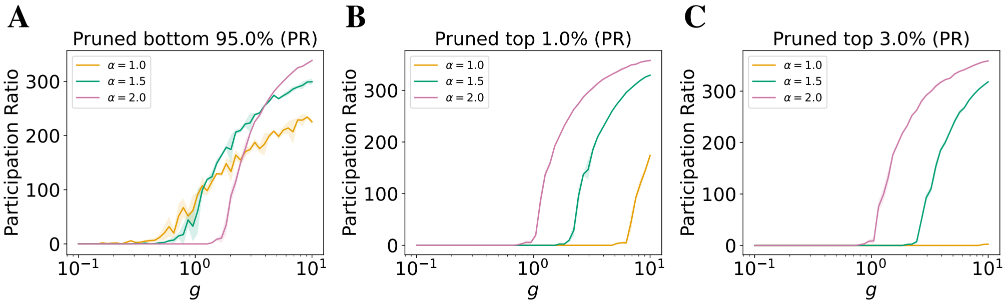

Figure 20: **Effect of pruning on participation ratio (PR).** (A) bottom 95% removed. (B) top 1% removed. (C) top 3% removed.

L Information processing in heavy-tailed recurrent neural networks

To examine whether our results extend to structured external inputs (and toward learned settings), we provide a proof-of-concept through a reservoir-computing experiment on the delayed-memory XOR task (a similar task is used in [Huh and Sejnowski, 2018]). We use networks of size $N = 1000$ and report average performance across three trials. Specifically, in the XOR task, each trial presents two binary stimulus vectors s_1, s_2 separated by silent delays; after the second delay, the readout must report $\mathrm{XOR}(s_1, s_2)$, requiring short-term maintenance of both stimuli and a nonlinear decision rule.

Across gains g, heavy-tailed reservoirs exhibited a broader and more stable operating regime than Gaussian reservoirs: the transition to chaos was slower and more robust (Fig. 21A), and task performance remained high over a wider range of g (Fig. 21B). These observations suggest that the extended critical regime of heavy-tailed networks can enhance robustness and performance without fine-tuning, with potential benefits for machine learning applications.

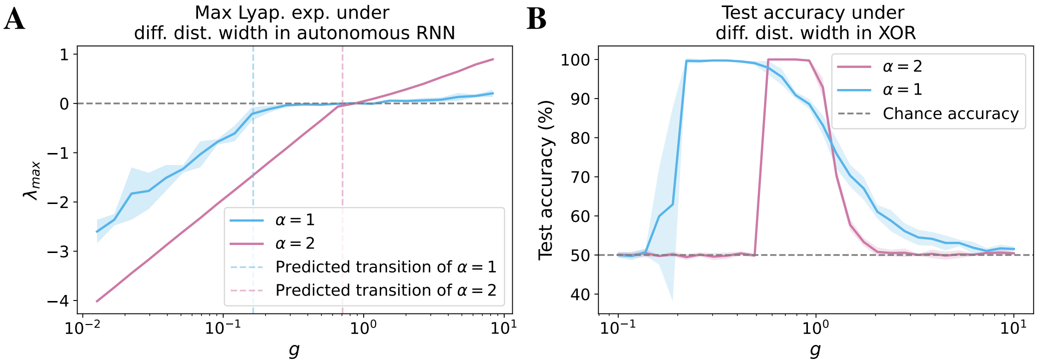

Figure 21: **Delayed-memory XOR with heavy-tailed reservoirs.** **(A)** Dynamics across gain g as measured by maximum Lyapunov exponent for Gaussian (pink) vs. heavy-tailed reservoirs (blue), showing a slower, more robust transition to chaos in the latter. **(B)** Task accuracy of a linear readout on the same reservoirs, with heavy-tailed networks maintaining strong performance over a broader range of g.

M Additional details

M.1 Experiments compute resources

All experiments reported in this paper can be reproduced using CPUs only; no GPUs are required. The only exception is Fig. 1, for which we strongly recommend using a single GPU to avoid potential compatibility issues with the JAX package. Jobs were executed on a compute cluster using a maximum of 4 CPU cores and 20 GB of memory per task (which is a very conservative allocation; for networks of size $N = 1000$, for example, 5 GB is likely sufficient). Each experimental run was allocated up to 24 hours of wall-clock time. Most runs completed well within this limit, with small networks $N = 1000$ usually completed within 5 hours running serially over a grid of 50 gain g values, three tail indices α, and over 3 trials. Storage requirements were modest and standard across all runs. While additional preliminary experiments were conducted during development, they did not require significantly more compute and are not reported in the final results.

M.2 Licenses for existing assets

This project makes use of several open-source Python packages. While the main paper does not formally cite each package, we acknowledge their use here and ensure full transparency by providing all code and dependencies in the released repository. Below we list each core package, its version, license, and citation if applicable:

**Package**	**Version**	**License**	**URL**	**Citation**
`jax`, `jaxlib`	v0.4.38	Apache 2.0	`https://github.com/google/jax`	[Bradbury et al., 2018]
`numpy`	v1.26.4	Modified BSD	`https://numpy.org/`	[Harris et al., 2020]
`scipy`	v1.15.2	BSD	`https://scipy.org/`	[Virtanen et al., 2020]
`torch`	v2.7.0	Modified BSD	`https://pytorch.org/`	[Paszke et al., 2019]
`tensorflow`, `keras`	v2.19.0, v3.9.2	Apache 2.0	`https://www.tensorflow.org/`	[Martín et al., 2015]
`matplotlib`	v3.10.1	PSF	`https://matplotlib.org/`	[Hunter, 2007]
`tqdm`	v4.67.1	MIT	`https://github.com/tqdm/tqdm`	–

Table 1: Third-party Python packages used in this paper, with version numbers, licenses, source URLs, and citations where applicable.

Python versions `>=3.10` and `<3.13` are recommended. All software dependencies are installable via `pip` using the provided `requirements.txt`. No proprietary assets were used in this study.
